# Exploration of micro-video teaching mode of college students using deep learning and human–computer interaction

**DOI:** 10.3389/fpsyg.2022.916021

**Published:** 2022-09-02

**Authors:** Yao Liu, Na Cai, Zizai Zhang, Hai Fu

**Affiliations:** ^1^School of Architecture and Urban Planning, Guangdong University of Technology, Guangzhou, China; ^2^Vocational Education Teachers Institute, Guangdong Polytechnic Normal University, Guangzhou, China; ^3^Hangzhou Preschool Teachers College, Zhejiang Normal University, Hangzhou, China; ^4^School of Humanities and Communication, Ningbo University, Ningbo, China

**Keywords:** deep learning, human-computer interaction, micro-video, interaction design, college students

## Abstract

In order to improve the efficiency of teaching and learning in Colleges and Universities (CAUs), this work combines the Browser/Server (B/S) framework with Model View Presenter (MVP) technology to build a college student–oriented micro-video teaching system based on Deep Learning (DL) and Human–Computer Interaction (HCI) technology. Firstly, it makes an in-depth analysis of the problems in the classroom teaching of Chinese CAUs. Three functional modules are designed for the micro-video online teaching platform: video management, user learning, and system management. Then, it uses MVP technology to analyze the use-cases of these three functional modules in detail. Based on this, the micro-video online teaching platform is designed using the B/S framework. The teaching platform interface layer realizes the HCI between the platform and users. The business logic layer responds to the user requests submitted and returns the processing results to the interface layer. Finally, the function test and stress test of each module of the micro-video online teaching platform is carried out. The test results show that the response time of the proposed micro-video teaching platform increases with the number of users. Under the peak concurrent users, the system response time is 6 s, without abnormalities. Meanwhile, the proposed teaching platform has improved students' satisfaction with classroom teaching by nearly 15% and improved the compactness of the college classroom by nearly 12%. When the number of virtual users increases and the number of services increases linearly, the Random Access Memory and Central Processing Unit growth rate is significantly lower than that of the number of services. These outcomes indicate that many system resources are reused, and the system has good scalability, which can meet users' needs for the network video teaching system. The proposed teaching platform provides a new idea for applying DL and HCI technology in researching college students' micro-video teaching mode.

## Introduction

The 21st century marks the advent of the micro era for human civilization. Various technologies entitled and featured by micro-level have entered people's life, such as microblog, micro chat, and microfilm. With technological development, information dissemination has been accelerated at its most since the birth of human civilization. The surging amount of information per unit of time has greatly shrunk the time for people to read and digest single information, which puts forward higher requirements for information structure that can attract the public in a shorter time and improve the public's interest in reading. Education, in particular, has also been greatly affected. Under the current educational system, teachers can hardly understand the learning situation of each student. Thus, how to help students learn more effectively and overcome learning difficulties has become the major concern of teachers, which can well be solved by the emerging of micro-courses (Arnold and Versluis, 2019; Li D., 2021). Micro-course learning is very advantageous in that it makes up for the problems of fewer class hours, nonrepetitive learning content, and lack of pertinence in traditional teaching patterns. Today, the development of micro-course is an improvement and innovation of the original learning and teaching patterns. This innovative way provides students and teachers with more opportunities to learn and show than ever before (Darling-Aduana, 2021; Wang et al., 2021). Most micro-courses, however, are conferred through micro-video resources, and learners only stay in the “specific experience” stage without interactive link design, namely, the interaction concept. By introducing micro-video materials into curriculum design, teaching can enrich the interaction between students–students and students–teachers, enhance the situational learning content and students' participation, further improve students' learning efficiency, and promote in-depth learning (Liang, 2019). Micro-video is slightly different from common video, which is short, brief, easy to be shared and transmitted.

Here, the Brower/Serve (B/S) framework in the deep learning and Human–Computer Interaction technology (HCT) is integrated with the Model-Presenter-View (MVP) technology to design three functional modules: video management, user learning, and system management of the proposed micro-video teaching platform. Finally, through the use-case analysis, B/S framework, the micro-video teaching platform is established for college students, thereby promoting the development of higher education.

The innovation of this work is that the proposed online micro-video teaching platform breaks the time and space constraints of traditional classroom teaching by extending the classroom time after class. It also provides a sound environment for autonomous learning and gives students more opportunities to participate in teaching and learning anytime and anywhere. Therefore, it fully reflects the teacher-led and student-centered teaching philosophy.

## Research status of online education and micro-course

### Research status of micro-course

Researchers have done abundant work on micro-course and online education. Specifically, Barabash et al. (2021), to solve the problem of the stability of the online education system, considered the comprehensive method of online education system structure and proposed to synthesize the network structure for distance education with the maximum functional stability criterion. The self-recovery tool of distributed software could be developed using the proposed composite model, and the characteristics of different computer resources could be considered using redundancy. The proposed method significantly reduced the recovery time of the system after or in case of failure. Wang et al. (2019) pointed out that most of the existing scientific and methodological means evaluated the operation quality of the distance education system through the effectiveness of its components and relevant quality indicators, which hindered the system from considering important factors in the decision-making. Therefore, the contribution of relevant subsystems to the operation of the distance education system should be fully considered. Given these problems, a comprehensive evaluation method was proposed for the effectiveness of the online education system based on probability theory and analytic hierarchy process, and the influence mode of the online education system was discussed. Li Y. (2021) suggested that with the popularization of Web 2.0 technology, students could actively learn and build personal knowledge networks. Research showed that 80% of knowledge was acquired outside the classroom, and the contribution of technology to education far exceeded the classroom projection and microphone. Therefore, the integration of such technology as Web 2.0 in university education would be the main direction of teaching. Thereupon, the author discussed the construction of the college English mixed teaching model based on modern educational technology and computer technology. Sergeev et al. (2020) put forward the problem of using videoconference services in the online education community under the background of educational space virtualization when organizing students' interaction during online learning. Then, the changes in the educational system discussed were related to the development of social networks and online communities, the new opportunities provided for teachers were analyzed to integrate students into various types of educational and cognitive activities, and the concepts of online community and social network were revealed. The results showed the role of network community and social network as the collective subject of social information and educational activities on the Internet. The research results have certain contribution to the theory of educational information and could be used for the practice of teaching using interactive services and Internet resources to implement the educational process with school-age children. Liu (2021) argued that the main purpose of the integration of network media and middle school ideological and political theory courses was to better realize the moral education function of middle school ideological and political development. Consequently, a multimedia resource integration system was proposed based on the wireless network for ideological and political remote teaching, which used XML (eXtensible Markup Language) to exchange data. The theoretical elements of design and the evaluation process of teaching resource management were clarified, and the automatic integration of teaching resources was realized. Finally, the subscription function of the dSPACE framework was invoked, and the subscription information was stored in the database. Whenever a new teaching resource was introduced into the subscription field, the dSPACE framework would automatically send an email to the subscriber.

### Summary and overview of research status

To sum up, the international research on micro-video has begun earlier than that in China, which focuses on the field of education and teaching, as well as the field of network information dissemination. The teaching field pays attention to the design of micro-video and its efficient utilization in teaching. The domestic research on the micro-course has started relatively late; and most of the current research focuses on the definition, characteristics, design, and development strategy of micro-course; while there is little research on the design and application of the integration of micro-courses and classroom teaching for specific disciplines, and there is little research on interactive micro-courses either.

## Analysis and implementation of micro-video teaching platform

### Related concepts about micro-video

When it comes to the definition of micro-video, there is no standard and unified statement but various explanations. “Micro-video refers to the general term for short videos that can be recorded and played by multi-channel video terminals, such as micro-film micro-documentaries, small Digital Video Format (DV) films or advertisements, with the shortest time of 30 s and the longest time of 20 min,” Gu Yongqiang, president of Youku, once explained. Micro-video is a new form of media that is different from single media such as text and pictures. It is a comprehensive media consisting of images, sounds, text, and other factors. Therefore, micro-video can give people a strong visual impact. It integrates the advantages of pictures, text, sound, and coexistence. Micro-video has the following three characteristics: (1) It is interactive. It is different from other traditional media, and it is not one-way transmission. Users can upload and download network micro-video, and micro-video viewers and publishers can exchange messages through the network. On the one hand, viewers give corresponding evaluations according to their own viewing effects at any time, which can increase the attention of micro-video; on the other hand, the creators can improve their work according to the feedback of the viewer, which well confirms the two-way interaction and good communication, which is conducive to the development of micro-video (Bogdanova, 2020; Ma and Li, 2021). (2) It has a visual impact. According to the famous French producer Deby, “the future world is the world of images, and the development of various industries will be inseparable from the direct or indirect help of images and videos.” There is evidence that vision and hearing play a vital role in people's sensory world, especially visual development is the most exquisite and effective. This also brings good enlightenment to educators. If teachers can make full use of the audio-visual effect of micro-video to carry out teaching, they will greatly stimulate students' enthusiasm for learning (Gerbaudo et al., 2021). (3) It has few limits. Different from professional film and television production, micro-video production has a low threshold and does not require producers to have high professional standards. Professional video producers must have a series of professional qualities as the background. They need to experience professional guidance. Furthermore, generally, these producers need to complete video projects together in the case of teamwork. However, micro-video production is very different. The level of micro-video production is uneven, which mostly belongs to personal behavior. The application of micro-video teaching resources has three significant advantages: (1) Micro-video has a strong visual impact effect. It is a favorable resource that can vividly present the content of physical education. The micro-video teaching method integrates pictures, sound, and text set in one, so that students can get full attention. It is very conducive to students' understanding and memory of knowledge and technology (Pham, 2022). (2) Micro-video has a strong density. It is usually short, but the content is very smart. According to psychologists' research, students' attention in the classroom is limited, and it is generally difficult to maintain full attention throughout the class. If teachers use micro-video for teaching, they can attract students' attention in a relatively short period of time and can basically achieve the purpose of conveying the key points and difficulties of the whole class to students in a short period, so that the learning effect is remarkable (Riordan, 2022). (3) Micro-video teaching resources can also enhance students' autonomous learning. Micro-video is a more flexible and practical sharing resources, students can also borrow the video from the teacher's courseware after class to watch and think repeatedly, and they are generally willing to accept the use of video resources for learning. Micro-video teaching resources then help students strengthen the impression of learning. Additionally, teachers and students fully interact with each other by watching and discussing the micro-video resources, so that the relationship between teachers and students is harmonious. In this process, students' learning initiative is improved, and their ability to explore knowledge is strengthened (Megawati and Trisnawati, 2022).

### Technology and demand analysis of micro-video teaching platform

**Figure 1:**
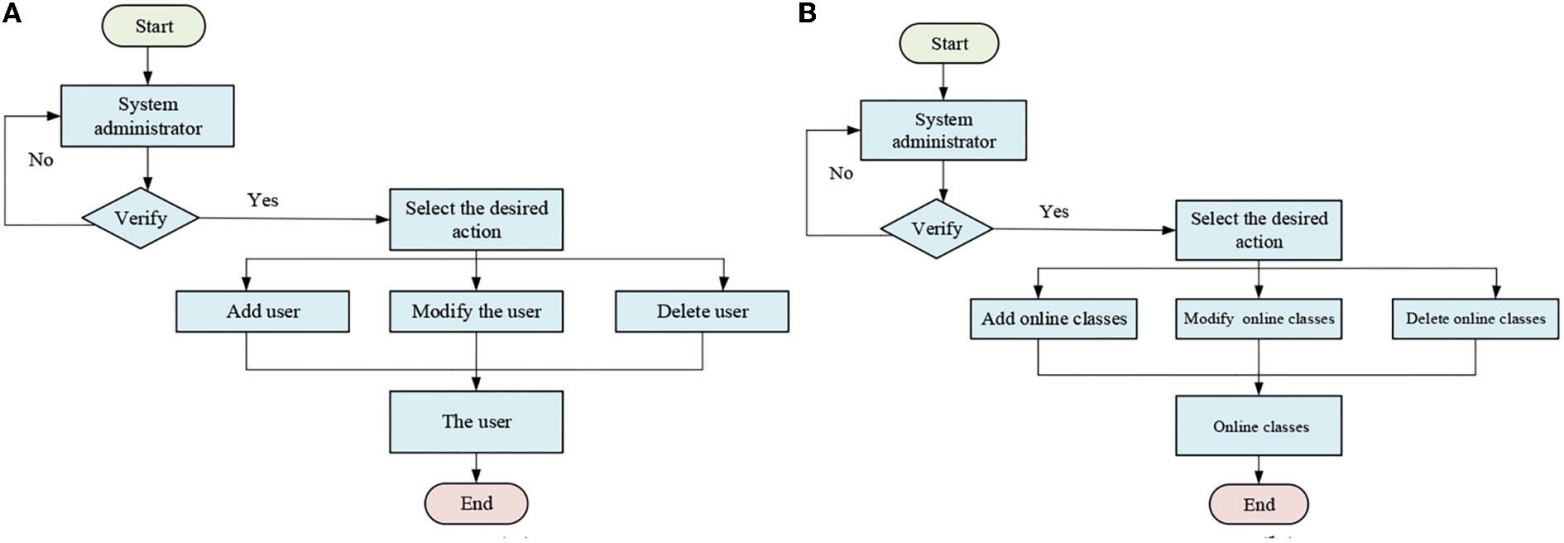
Business flow diagram. **(A)** Flow diagram of users management of the system. **(B)** Flow diagram of online courses management of the system).

The user of the micro-video teaching mode designed here is a sophomore, majoring in education in a college. The student has a clear purpose for learning. His learning time is under a certain guarantee, and he has the skills and ability to conduct micro-video learning. Most students are not taught by micro-video teaching mode. This work uses the micro-video in the form of streaming media converted from Flash, PowerPoint (PPT), and other resources to study. According to the actual needs of teaching process, the system developed in the process needs to meet the basic needs of system practicability, system reliability, and system scalability (Chen, 2019). Users of micro-video teaching platforms are divided into three types: The first is the system administrator, the second is responsible for uploading teaching micro-video uploading, and the third is the student user, who logs in teaching platform to watch micro-video. According to the actual needs of the course, the system needs to be implemented including functional modules such as system user management function, message function, quality assessment function, classroom investigation function, and online classroom function module (Lin and Chen, 2020). The teaching auxiliary system enhances the interaction between teachers and students. The problems in the teaching process can be timely feedback, to improve the teaching quality. The advanced technology is used to build a network-based curriculum teaching auxiliary system, which is intended to be an online teaching auxiliary system and an interactive platform between teachers and students. The online teaching auxiliary system mainly includes the following business (Rogoza et al., 2018): (1) System user management: Its main functions are to create, query, delete system users, and assign corresponding permissions to system users. (2) Message management: Its main functions are to add, modify, and delete message information. Besides, it functions in auditing and replying to the message information of system users. (3) Certification registration management: Its main function is to add information about students and modify the certification registration information. System administrators can use it to audit the registration information. (4) Quality assessment management: Its main functions are to add, query, modify, and delete quality assessment information. (5) Courses investigation management: Its main functions are to add, modify, and delete classroom investigation information. (6) Online courses management: Its main functions are to add, modify, and delete online classroom information. In the process of analysis, initially, the business flow diagram is used to describe the process processing in a specific business process. The following two main business flow diagrams are selected for analysis. Figure 1 shows the business flow diagram of the system.

### Requirement analysis of functions

**Figure 2:**
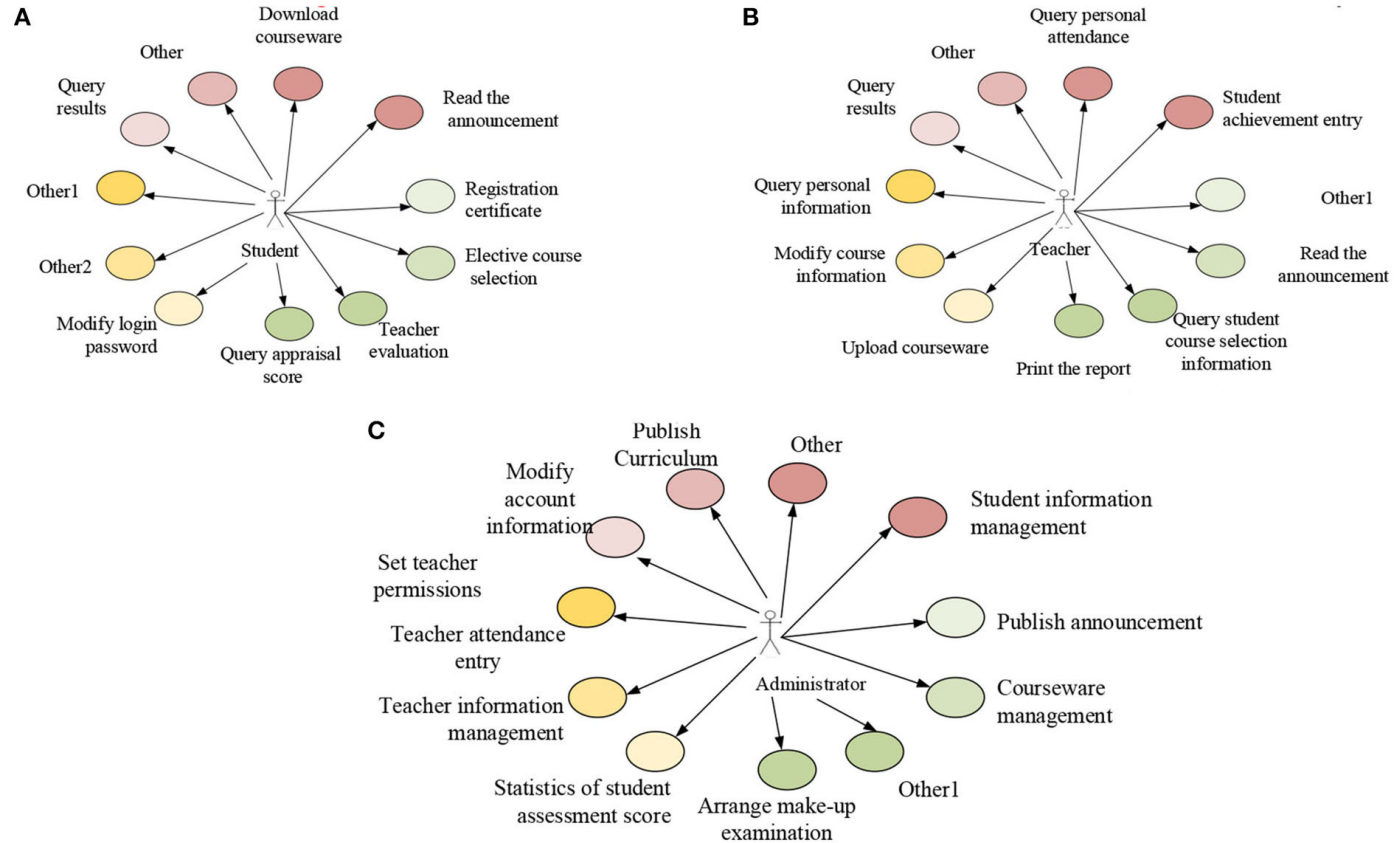
Use case diagram. **(A)** Use case analysis of students. **(B)** Use case analysis of teachers. **(C)** Use case analysis of administrators.

The use case is an important functional unit in the system. It is a description of the function provided by the system and the interaction process between participants and the system. Participants are people or external systems using use cases. A use-case diagram describes use cases and participants in the system from the perspective of participants, including the relationship between use cases and participants. Initially, a description is conducted of the overall function of the system. The online micro-video teaching system is divided into three main users by analyzing the participants. They are the teacher who manages the online course, the student who uses the course for learning, and the system administrator user who manages the system as a whole. The system users are analyzed using the use-case United Modeling Language (UML) technology. Figure 2 demonstrates the top use-case diagram of the system.

Teachers are mainly responsible for course management in the online micro-video teaching system. Online course management provides functions, such as uploading and maintaining course files. The course videos are stored in the memory as files. Therefore, the network video course is mainly managed as a file. Students are the main users of the online video courses to complete learning by browsing and searching their desired content from many files and can evaluate their learning effect and practice accordingly. Meanwhile, they can subscribe to specific courses. The system administrator manages the teaching platform, including user and comment information management, among others. Besides, administrators will review the online video courses submitted by teachers and delete the unqualified courses. The administrator will high-rate the teaching resources to recommend to students. Other works of an administrator include teacher–student information management and evaluation information in the system.

The system mainly consists of five functional modules: user management, message management, quality assessment management, courses investigation management, and online courses management. The following describes the function of each functional module in detail and summarizes the main functions. The user management module: This module is a system used to manage the current users uniformly, such as adding new management users, viewing administrator information, modifying the login password of management personnel, and launching a user management interface to return to other management windows. For the sake of system security, the administrator's last login address and the last login time can be displayed in the view administrator information function. For the main use cases in the use-case diagram of the system user management module, the use cases are described in the specified table of the use case, as listed in Table 1.

**Table 1:** Use case table for “users management”.

**Cases**	**Description**
Name of the use case	Adding new users
Statement of the use case	Accomplishing the statement of the user
Participants	Administrators of the system
Pre-conditions	Administrators entering the interface of “users management”
Post-conditions	The system noticing administrators that the users' information has been successfully input
Basic operating flow	1. Administrators enter the interface of inputting users' information 2. Administrators submit the input information 3. System notices administrators that the users' information has been successfully input
Included use case	Users management

Message management module: The module is a platform for interaction between students and teachers or between students and courses. The message module function shows the time and address information of the message. If the administrator has no problems with the messages after the audit, the professional teacher answers the message one by one. Besides, only the message that answers the accorded information can be displayed normally. Other messages are temporarily blocked and are allowed to be displayed after getting the correct answer. The use-case diagram of the message management module in the system is responsible for the interaction between students and teachers or between students and courses, including adding administrator mailboxes, modifying administrator information, viewing administrator information, and deleting administrator information. Quality assessment management module: The evaluation of teaching quality aims to check teachers' implementation of teaching norms and completion of teaching tasks, helps teachers diagnose and improve teaching, and provides reference for relevant departments to make decisions. The quality evaluation of the curriculum auxiliary system is managed through multifaceted evaluation of off-campus experts, on-campus teachers, and on-campus students. Classroom investigation management module: The learning effect of the course is obtained by an anonymous survey.

### Analysis of related technologies

**Figure 3:**
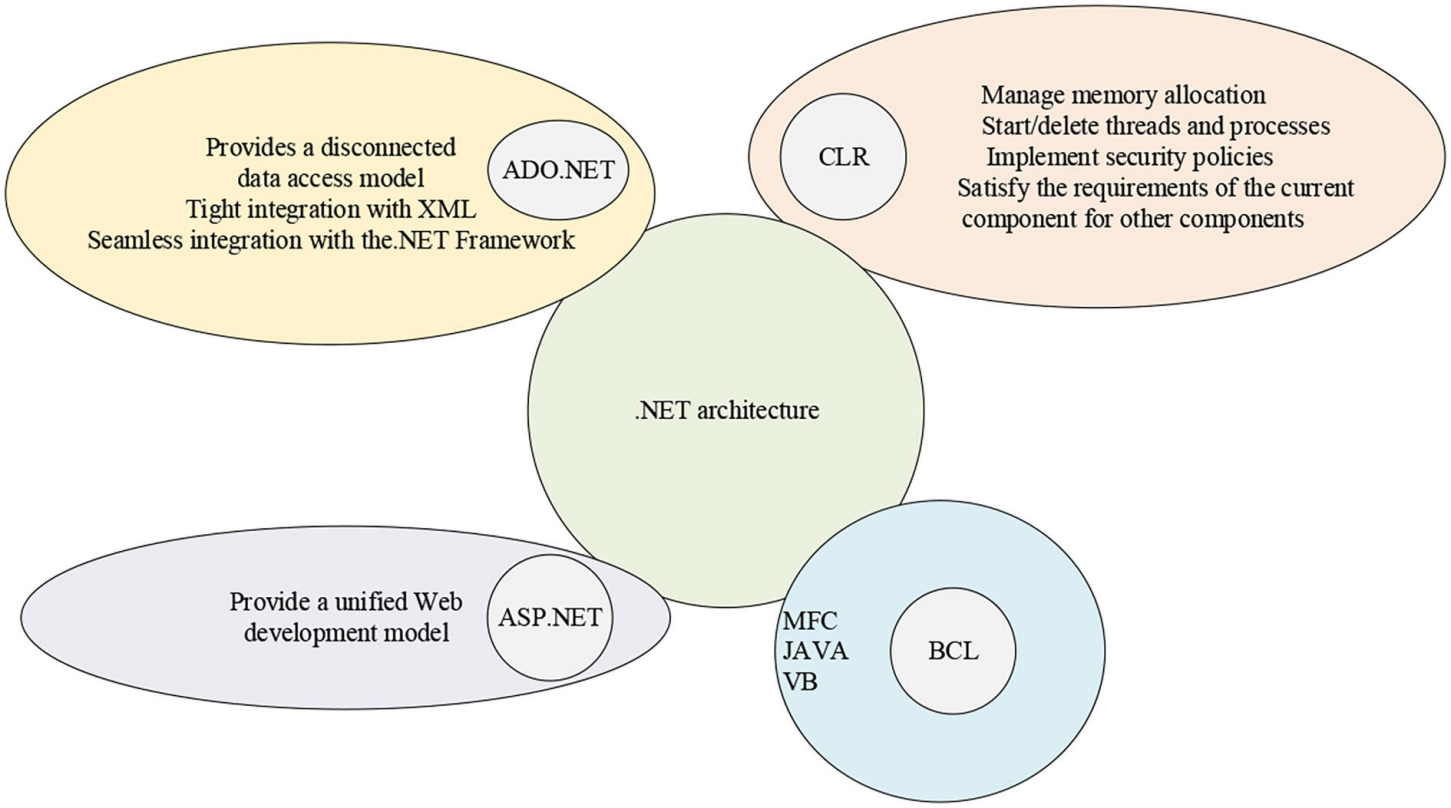
.NET framework architecture.

.NET framework runs on the Operating System (OS) and includes common language runtime and framework class library. The .NET framework can provide a unified environment across programming languages. Framework Class Library (FCL) is a comprehensive object-oriented reusable typeset, which can develop traditional command-line applications, WinForms applications, and ASP.NET-based applications. From the perspective of software design (Cinganotto, 2019), the .NET framework architecture is shown in Figure 3.

**Figure 4:**
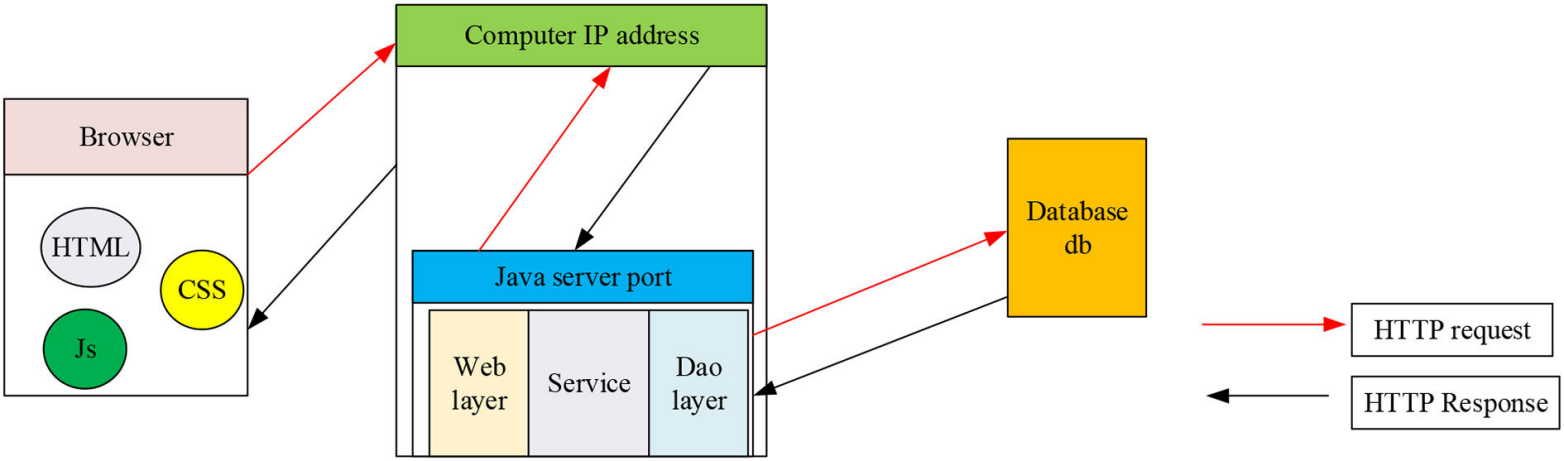
B/S structure diagram.

A software design with information interaction should consider the interaction among users and systems, user requests and responses, and data storage management (Mccredie and Kurtz, 2019; Yuan and Wu, 2020). The B/S mode is short for the browser/server mode. Under the system development through the B/S mode, the application layer is generally deployed to the server-side to facilitate the server to process the user's requests more efficiently and to achieve more complex functions. The Web program is responsible for receiving the requests of users and system administrators and can use the server to display the processing results of the requests on the browser interface. The B/S architecture adopts the working mode of browser request and server response (Goh et al., 2020). The working principle of B/S architecture is shown in Figure 4.

Figure 4 shows that the workflow of B/S architecture follows four steps: (1) Client sends requests. (2) Server-side processes requests, server-side receives request information, and server-side processes request data using Java Server Pages (JSP). (3) The server sends a response, that is, the server returns the data requested by the user to the browser. (4) The browser interprets HyperText Markup Language (HTML) files and presents them on the user interface (Zheng et al., 2018; Wu and Song, 2019).

**Figure 5:**
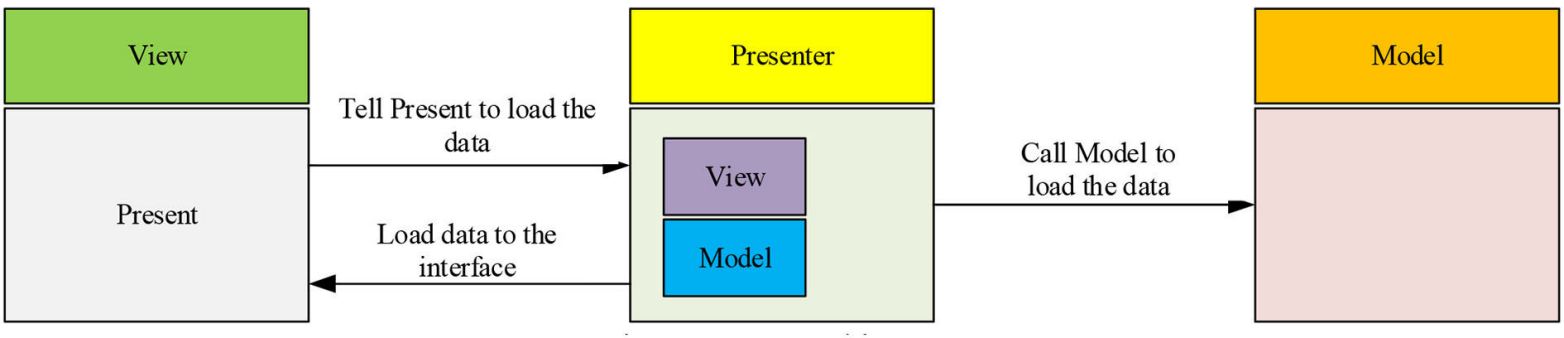
MVP architecture.

The relationship between the system object categories is very complex. Here, the Model-View-Presenter (MVP) (Liu et al., 2019; Onorato et al., 2019; Wu et al., 2019, 2020) pattern with the data layer, business layer, and the presentation layer is combined with the B/S pattern to design the teaching system architecture. Meanwhile, the JSP technology is chosen for visualization control (Railean et al., 2021). The MVP architecture diagram is shown in Figure 5.

The online micro-video teaching system includes a server and a client. The client can be a teacher or student computer and collects users' video and audio multimedia data, while the server transmits and stores these data. The server connects the client and the core of the whole teaching system. Thus, the server performance directly affects the whole system performance. The login control module can authenticate the user authority by only allowing authorized users to access the online micro-video teaching system.

Further, the client is divided into the student and teacher sides. There might be some differences in specific functions, but the overall functions that the two sides want are similar, mainly including four functions: (1) *Login control:* Only authorized users by the login control module can receive the Internet Protocol (IP) multicast data sent by the server-side and access the relevant system functions. (2) *Data collection:* Students are required to collect local multimedia information through the collection equipment and multicast it to the corresponding client through the server for display. The client collects the audio and video multimedia data mainly through the camera and microphone and encoded by the codec module. Then, the data are transmitted to the server for multicast. (3) *Data encoding/decoding module:* The multimedia data have a large volume. Thus, limited by the existing network technology, the transmission of these data will reduce the network performance. Therefore, the audio and video coding/decoding module can compress the multimedia data to improve the system performance. At the same time, the client must decode the multimedia data from the server before playing them with the Java Media Framework (JMF) player. (4) *Data playback:* The client decodes the multimedia data and uses the JMF player to play the multimedia data multicast by the server.

### Implementation of micro-video teaching platform

**Figure 6:**
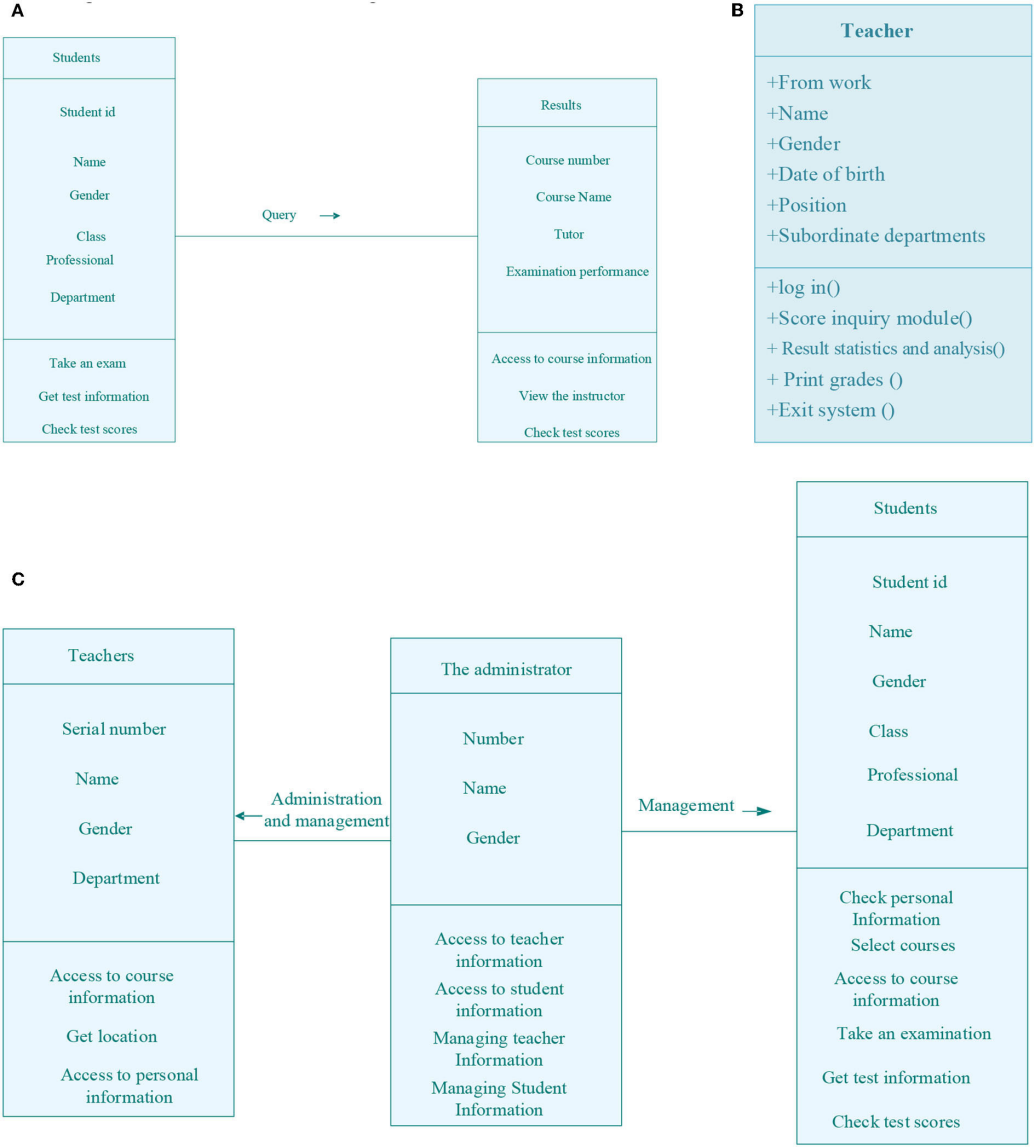
Schematic diagram of micro-video management function. **(A)** Class diagram of students' achievements management; **(B)** Class diagram of teachers' information; **(C)** Class diagram of administrator management.

An object class diagram can be drawn according to the defined object class and its relationship, the role, multiplicity, navigation, and other properties of the object class. Figure 6A refers to the class diagram of students' achievement management. Figure 6B indicates the class diagram of teachers' information. Figure 6C describes the class diagram of administrators' management information.

### Realization of the micro-video teaching platform

**Figure 7:**
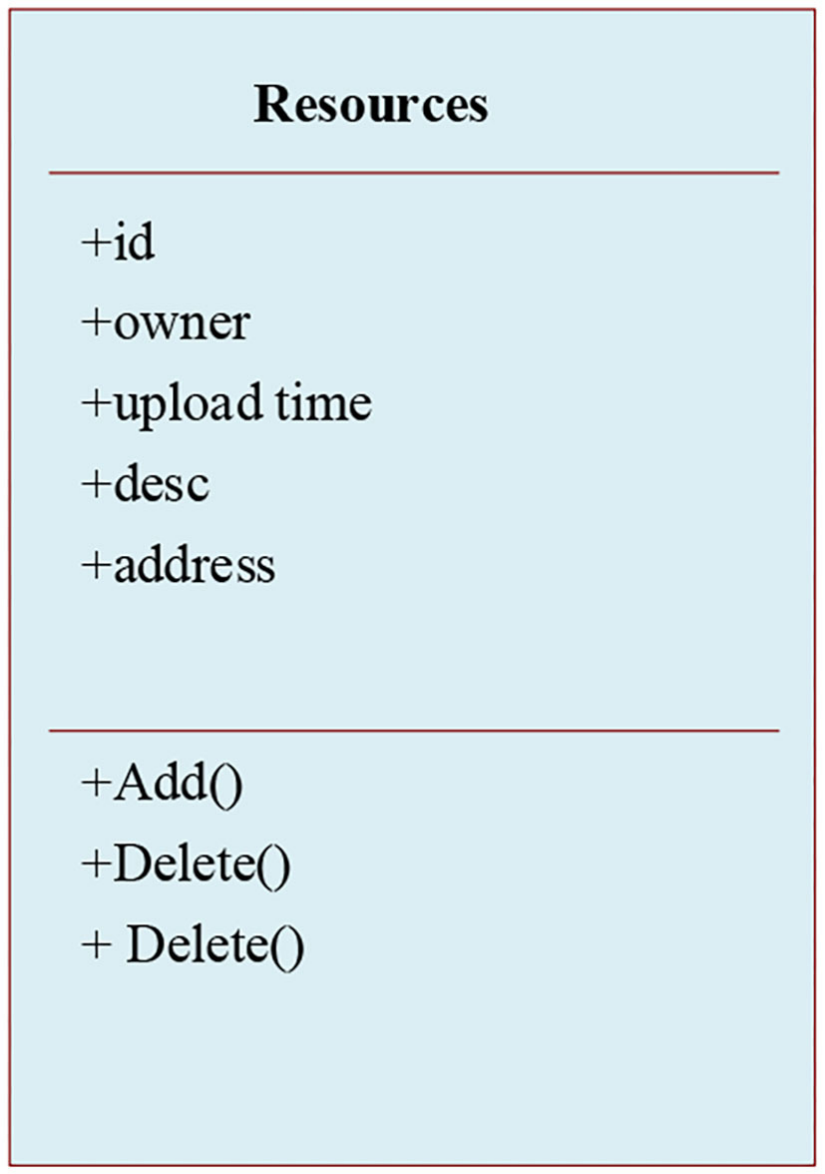
Class diagram of micro-video management function.

The micro-video management function mainly includes a series of basic operations such as uploading, updating, querying, and deleting experimental micro-video. Figure 7 illustrates the class diagram of micro-video management function.

**Figure 8:**
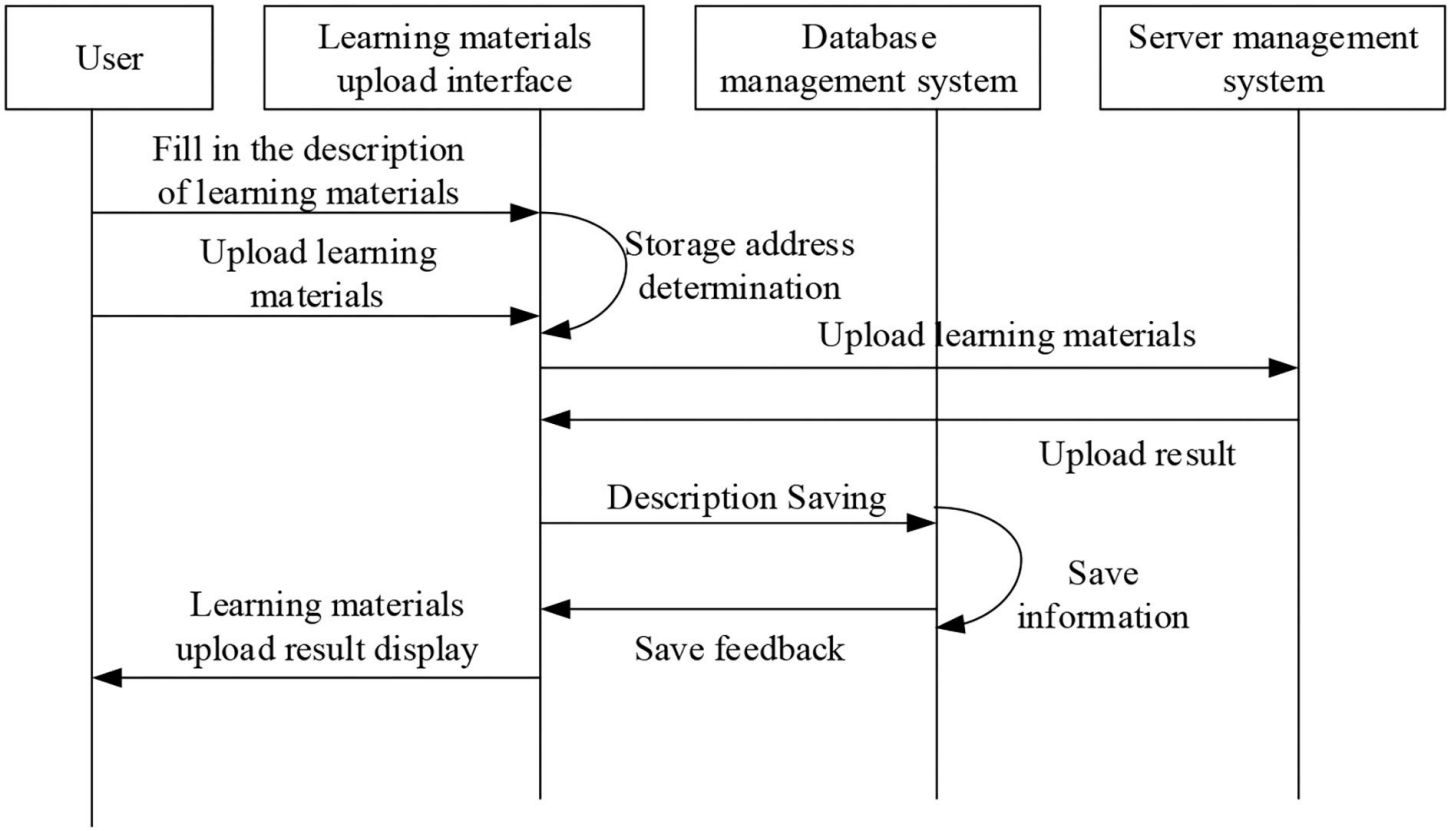
Sequence diagram of micro-video course upload.

**Figure 9:**
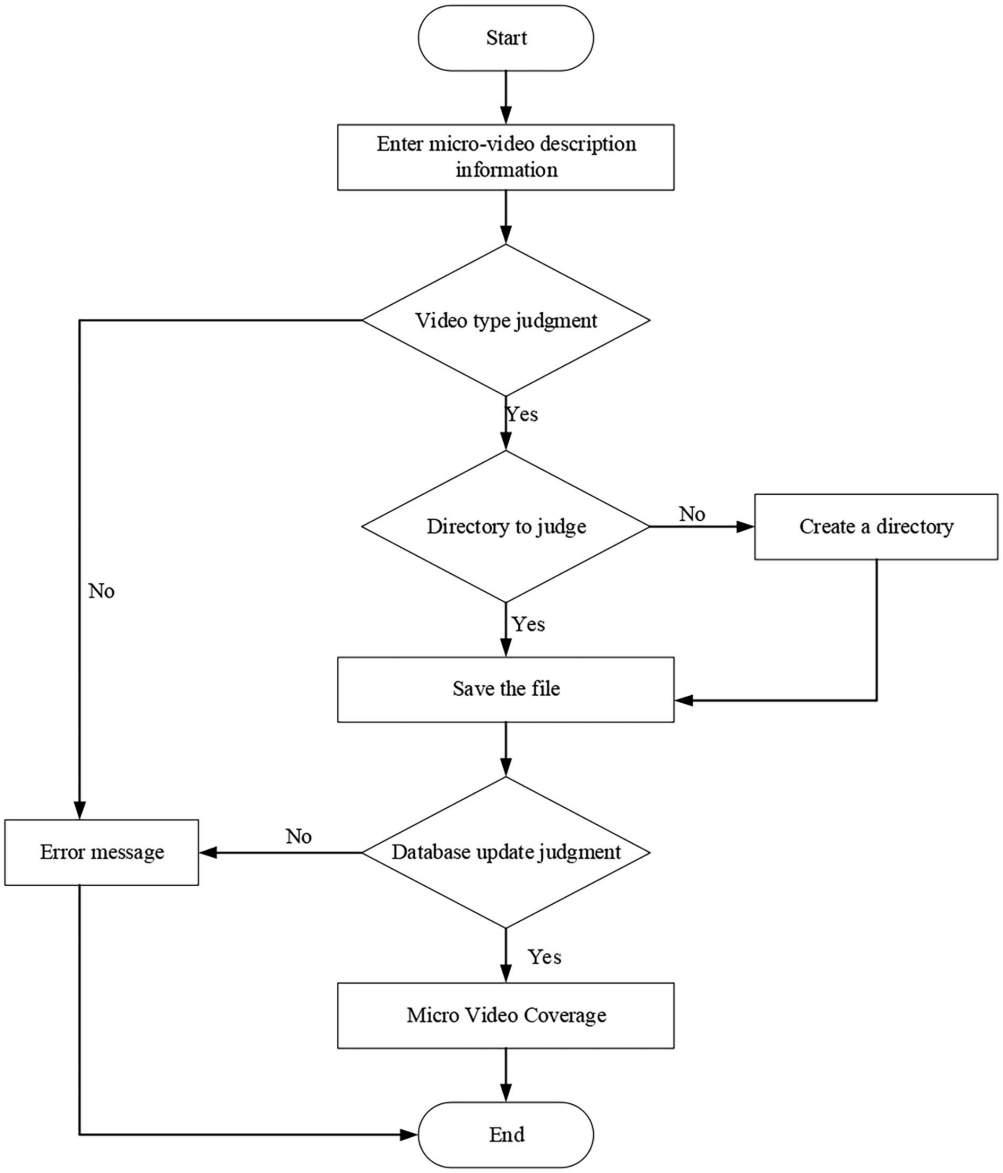
Micro-video upload flowchart.

Figure 7 illustrates that the class of micro-video management function mainly saves the text description information from teacher users for uploading experimental micro-video, and the corresponding database operation function. The sequence diagram of micro-video course upload is shown in Figure 8, and the flowchart of micro-video upload is shown in Figure 9.

**Figure 10:**
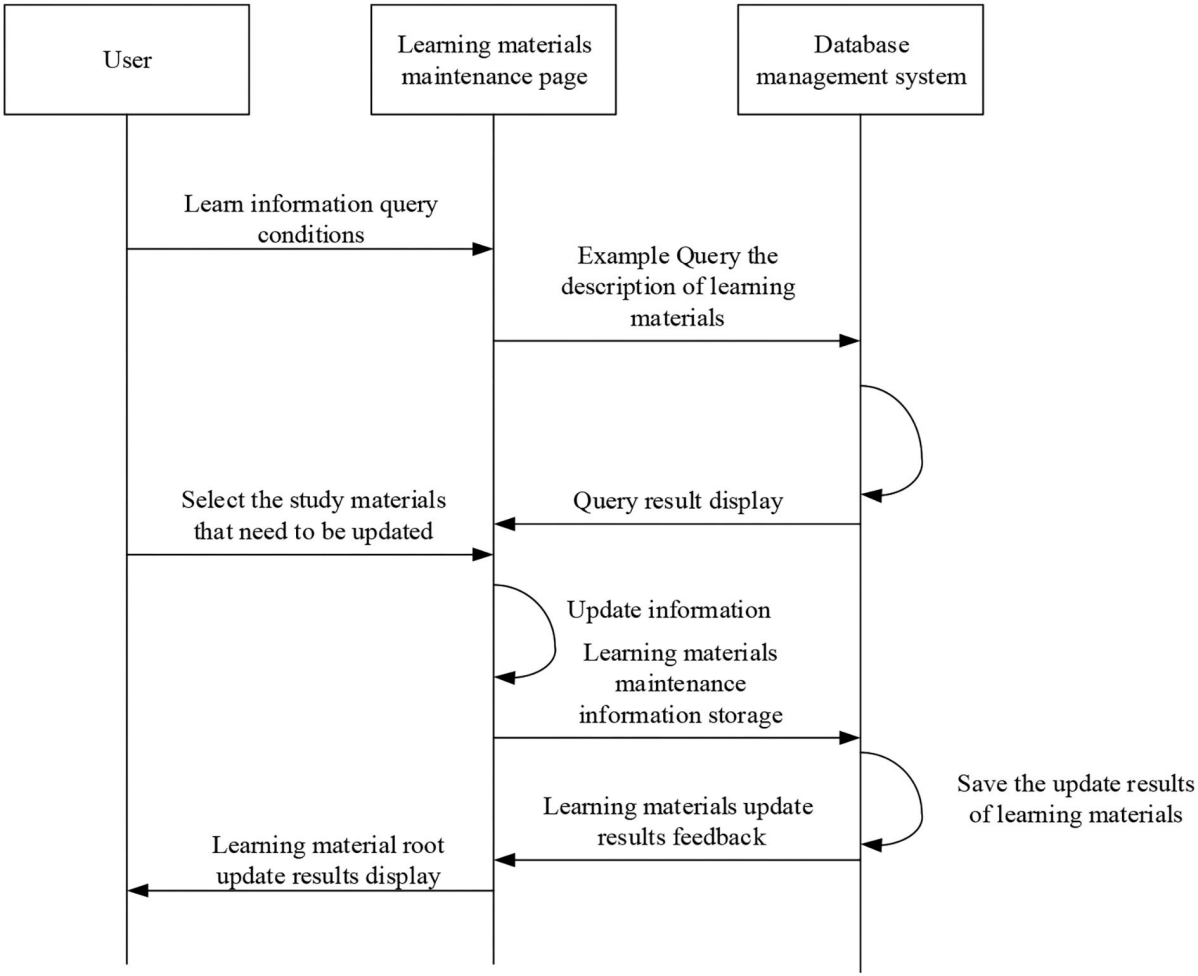
Sequence diagram for the micro-video course update.

After logging in, teachers can create new courses on the micro-video teaching platform. After the new course is completed, the physics experiment micro-video made by the teacher on the local computer is uploaded with a click on the corresponding button to the created course and saved to the database of the platform. Similarly, the micro-video course description written by the teacher is uploaded to the created course and saved. In the upload micro-video filling interface (Chen et al., 2020; Feng and Chen, 2020), the teacher can fill in the information description of the micro-video course and finally, complete the upload of the course micro-video. Teachers can display corresponding course information on the query interface and query the micro-video course library in the teaching platform (Li L. H., 2020; Liu et al., 2021). The micro-video update sequence diagram is shown in Figure 10.

**Figure 11:**
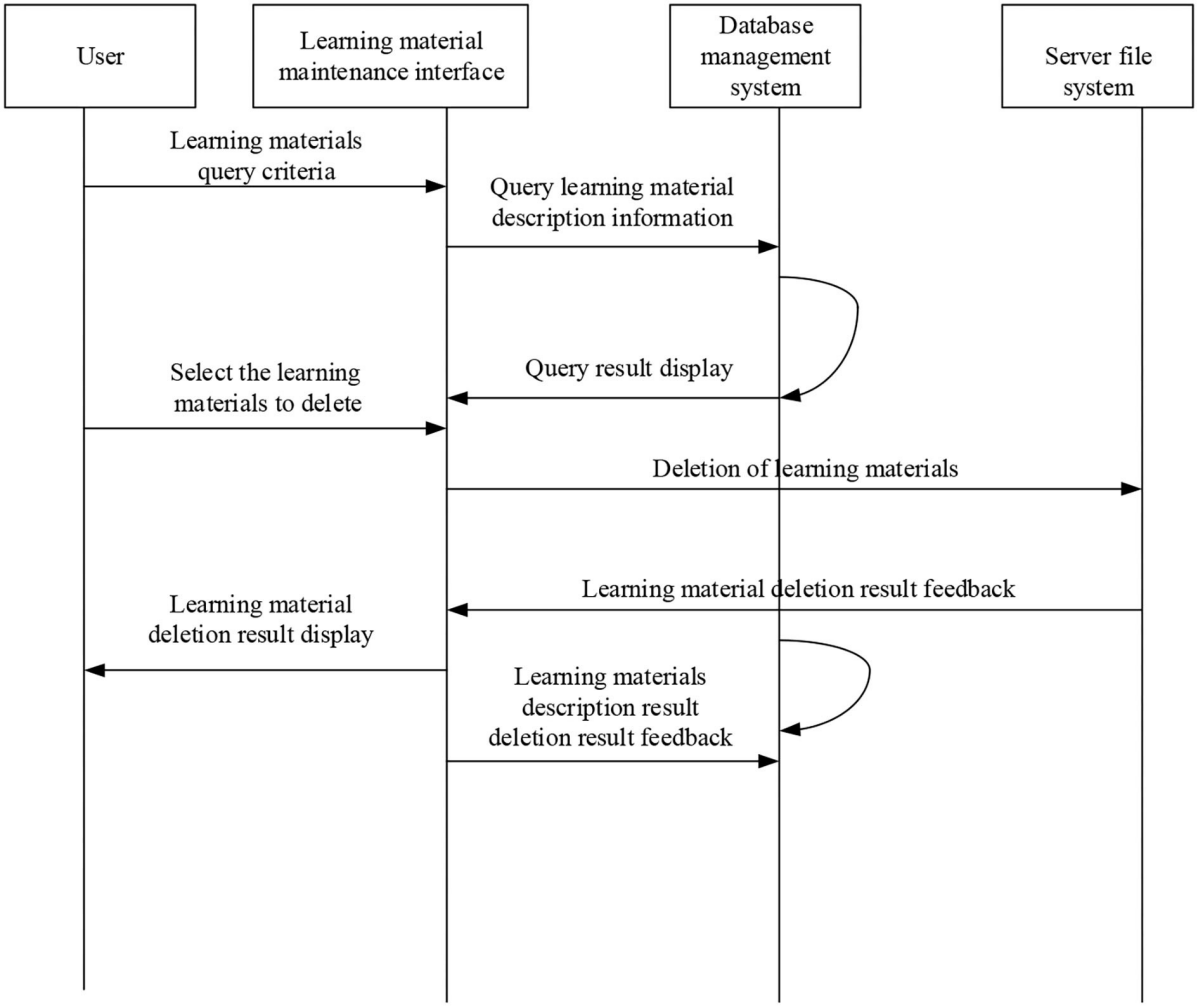
Sequence diagram of micro-video deletion.

**Figure 12:**
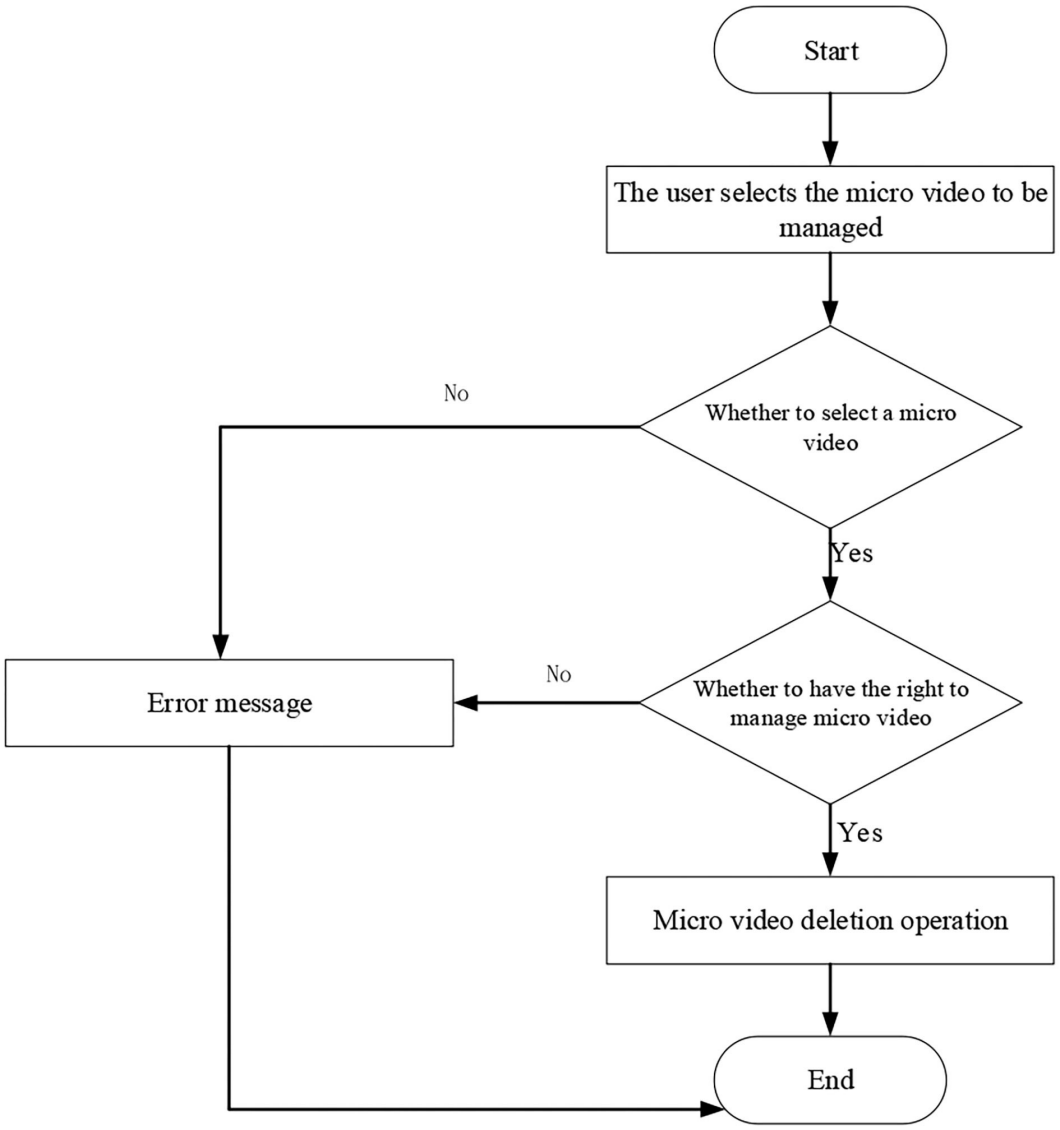
Flowchart of micro-video deletion.

Figure 10 implies that for the micro-video course update, the original micro-video should be deleted first, and then the micro-video can be uploaded. For a teacher to upload micro-video, files of the micro-video course are deleted first, and then the corresponding descriptions are deleted (Deng et al., 2021). The sequence diagram of micro-video deletion is shown in Figure 11, and the flowchart of micro-video deletion is shown in Figure 12.

**Figure 13:**
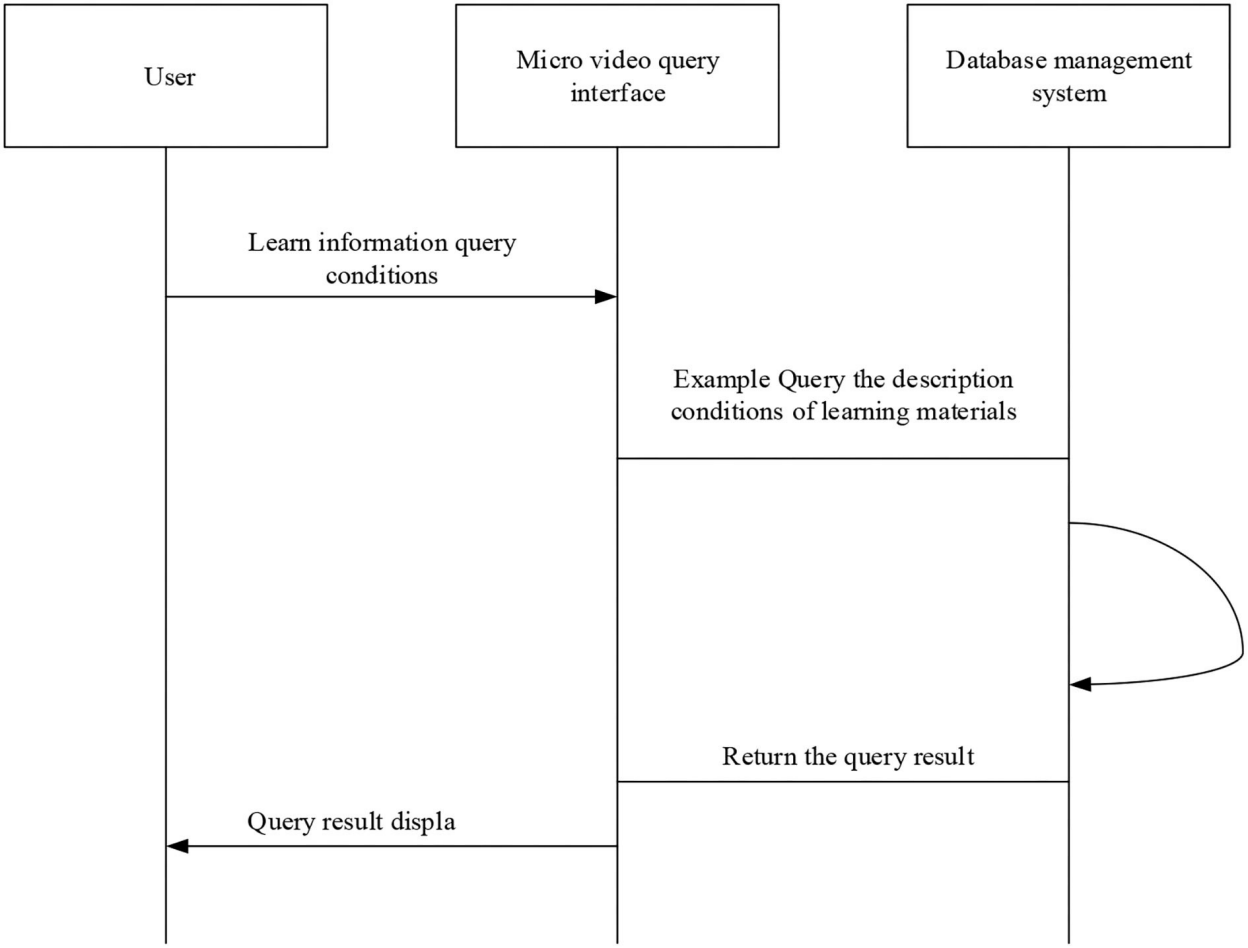
Sequence diagram of the micro-video query.

The use-case analysis of micro-video students indicates that during micro-video learning, students can perform three operations: query of micro-video course, download of the target micro-video, and evaluation of micro-video course. The sequence diagram of the micro-video query is shown in Figure 13.

**Figure 14:**
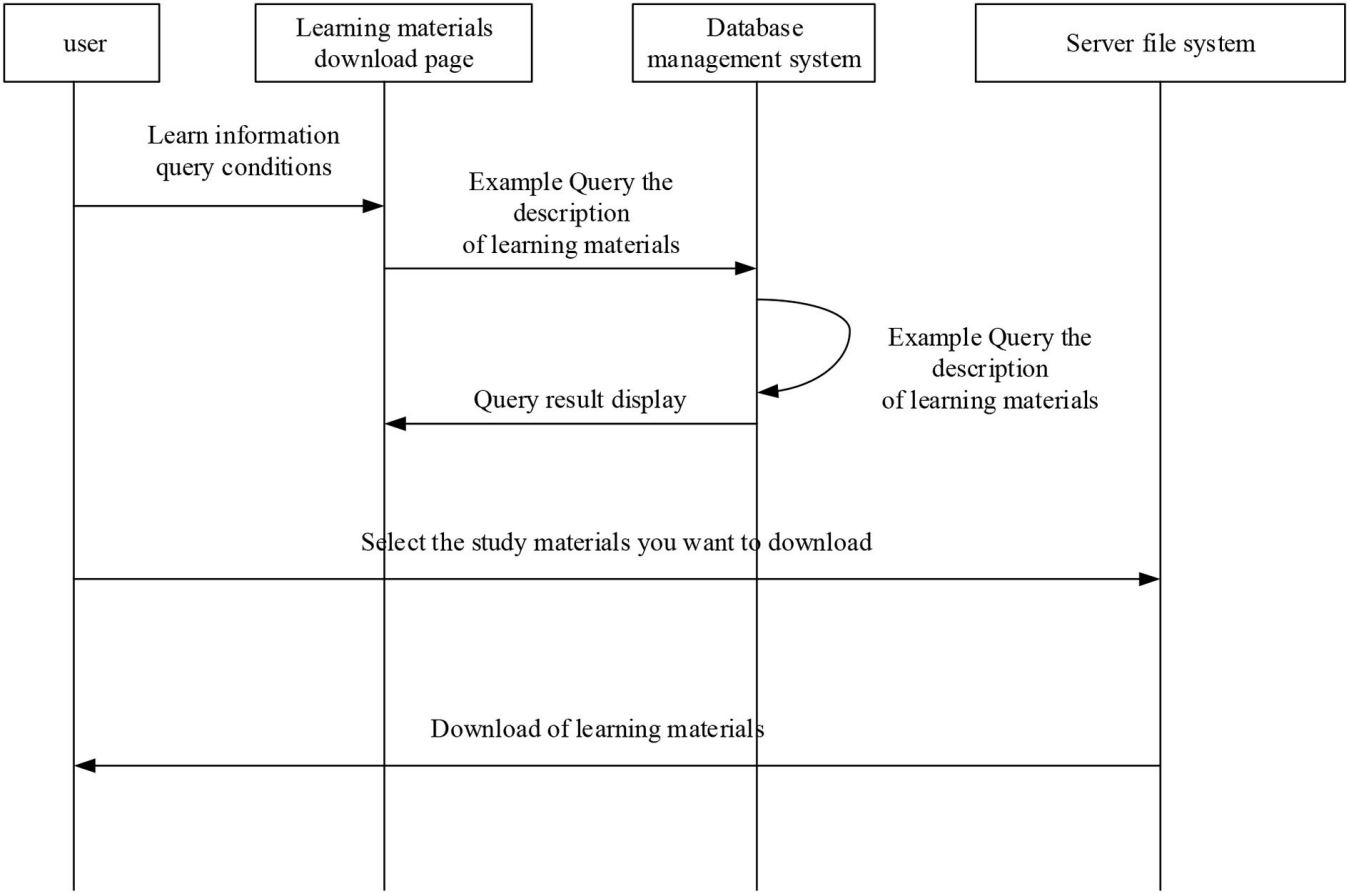
Sequence diagram of the micro-video download.

First, the users enter their account name and password and successfully log in to the teaching platform. Then, they input the query information in the information query window of the system interface, and the system transmits the query information to the background. The background matches the user's query conditions with the teacher's course description on the micro-video courses. Finally, the requested micro-video course is presented on the software interface for students to watch and learn. The sequence diagram of micro-video download is shown in Figure 14.

**Figure 15:**
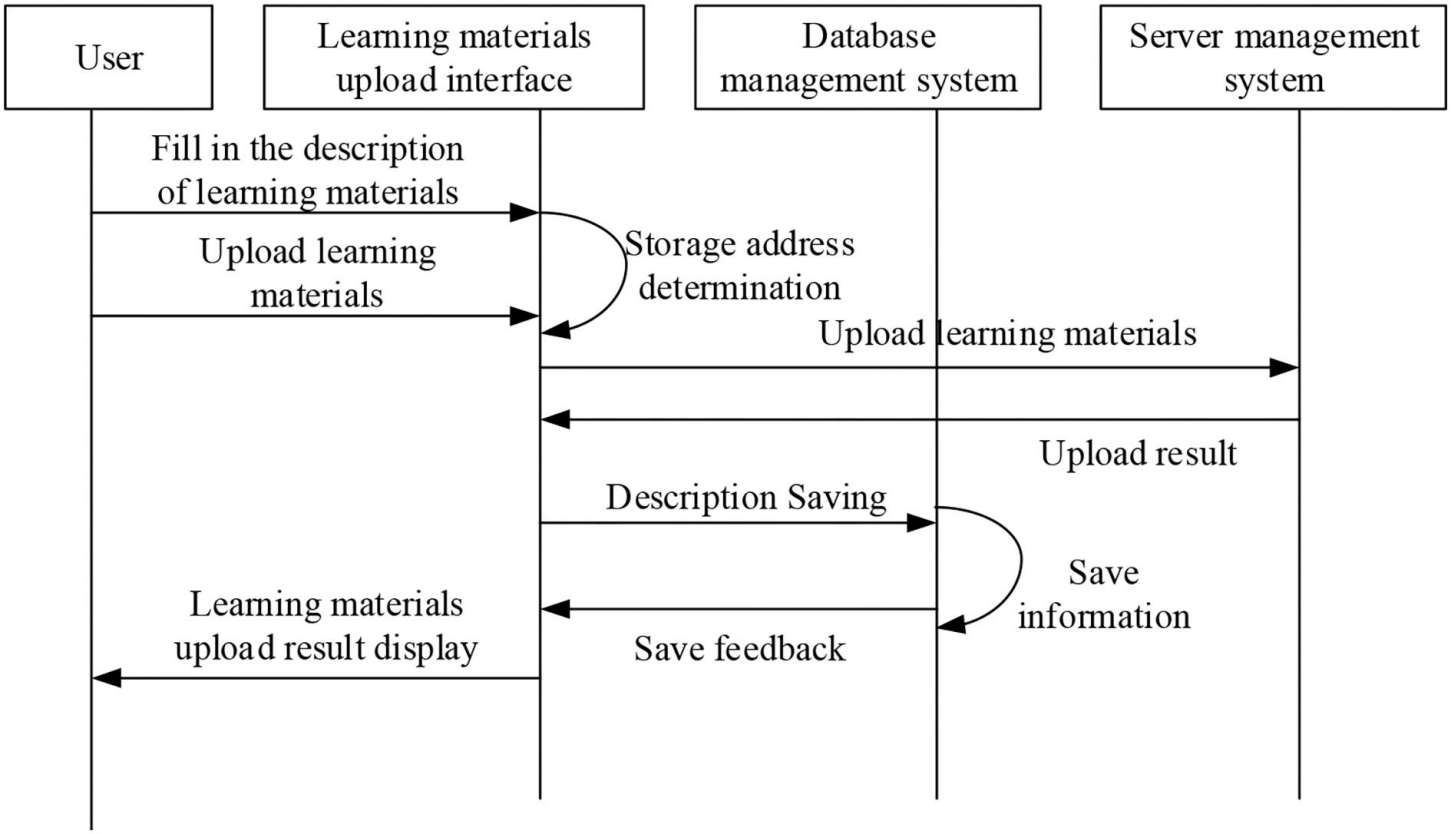
Sequence diagram of the micro-video evaluation.

Figure 14 suggests that students can download their demanded micro-video course files using HyperText Transfer Protocol (HTTP). Besides, the teaching system supports micro-video download to PC, after which students can use self-learn relevant courses, or students can choose to log in to the platform for online learning. The micro-video teaching platform is also designed with the evaluation function of the micro-video courses, based upon which students can learn according to their learning effect and the quality of the micro-video courses. The sequence diagram of micro-video evaluation is shown in Figure 15.

In summary, the system designed here is divided into an authority management module, curriculum management module, knowledge point management module, topic management module, interactive learning module, interactive practice module, interactive evaluation module, and data reporting module, totally eight modules. The role of the authority management module is to allocate and manage the authority of accessing system resource and function to the two groups of curriculum administrators and users separately. The function of the curriculum management module is to manage the external framework, directory tree, and teaching content of curriculums. The function of knowledge point management module is to maintain the knowledge point content, knowledge point directory, and the relationship between knowledge points. The topic management module completes the topic editing and entry, and the topic display style control. The interactive learning module presents the teaching content to students through the material form. The interactive practice module is the core module of the system. Students complete the interactive practice and submit the answer. The system sets up data acquisition points in this module to collect question information and interactive records and to complete statistics in the backstage terminal. The interactive practice module is used to consolidate the learning results of student in the learning process. The interactive evaluation module in the system mainly meets the needs of students to simulate the test, restore the test scene, and comprehensively analyzes the current level of students through the statistics of test scores and learning behavior data, so as to help students optimize their learning programs based on curriculum learning objectives. The main function of the data reporting business module is to comprehensively organize and display the learning information of students in the formulation period. By sorting out all kinds of learning data, the learning plan can be adjusted in time in the target-oriented teaching system to ensure that the learning content is balanced and comprehended in the expected time.

### Construction of performance evaluation model for micro-video teaching platform

The teaching quality evaluation model can be divided into two parts: predicting students' academic performance based on multilayer Backpropagation Neural Network (BPNN) and the statistical analysis of users' learning emotions based on micro-expression recognition. In particular, BPNN is a multilayer forward network with input, hidden, and output layers. The input layer receives signals, the hidden layer implicitly transforms signals, and the output layer is responsible for outputting the prediction results. The basic structure of BPNN is depicted in Supplementary Figure S1.

In Supplementary Figure S1, X, Y, and Z represent the number of nodes in the input, hidden, and output layers, respectively. {*w*_11_, *w*_12_, …*w*_*nl*_} means the weight between the input-layer node and the hidden-layer node. {*v*_11_, *v*_12_, …*v*_*bn*_} denotes the weight between the hidden-layer node and the output-layer node. {*b*_1_, *b*_2_, …*b*_*m*_} stands for the threshold of each output node. BPNN mainly uses the Gradient Descent Method (GDM) of the error function to learn and continuously circulate the input signal's forward propagation. Simultaneously, the error reverse propagation continues until the required error is met. Only then the algorithm stops.

Principal Component Analysis (PCA)-Back Propagation (BP) method uses PCA to reduce the data dimension and the dimension of evaluation indicators and eliminates the correlation between data while ensuring the minimum loss of system information. Most of the data are extracted by retaining the principal component information with a contribution rate of more than 90%. The output of PCA is used as the input of BPNN. Then, the BPNN is trained and verified by 35 groups of data, and ten groups of data test the trained BPNN model.

The correlation coefficient matrix of the experimental data can be written as in Equation (1):


(1) $$R=\left|\begin{array}{cccc} r_{11} & r_{12} & \cdots & r_{1p}\\ r_{21} & r_{22} & \cdots & r_{2p}\\ \cdots & \cdots & \cdots & \cdots \\ r_{p1} & r_{p2} & \cdots & r_{pp} \end{array}\right|$$

In Equation (1), *r*_*ij*_ is the correlation coefficient between the original variable *x*_*i*_ and *x*_*j*_ and can be expressed by Equation (2):


(2) $$\begin{array}{r} r_{ij}=\frac{\sum_{k=1}^{n}\left(a_{ki}-{\bar{a}}_{i}\right)\left(a_{kj}-{\bar{a}}_{j}\right)}{\sqrt{\sum_{k=1}^{n}{\left(a_{ki}-{\bar{a}}_{i}\right)}^{2}{\left(a_{kj}-{\bar{a}}_{j}\right)}^{2}}} \end{array}$$

In Equation (2), $i=1,2,\cdots p,j=1,2,\cdots p,{\bar{a}}_{i}=\frac{1}{n}\sum_{k=1}^{n}a_{ki}$.

The principal component contribution rate reads:


(3) $$\begin{array}{r} \frac{\lambda_{i}}{\sum_{k=1}^{p}\lambda_{k}},\;i=1,2,\cdots \text{p} \end{array}$$

In Equation (3), **λ**_***k***_ is the vector obtained by arranging the eigenvalues from large to small.

The cumulative contribution rate of information reads:


(4) $$\begin{array}{r} \frac{\sum_{\text{i}=1}^{\lambda_{\text{i}}}\lambda_{i}}{\sum_{\text{k}=1}^{p}\lambda_{k}},\;i=1,2,\cdots \text{p} \end{array}$$

Then, the principal components corresponding to the eigenvalues with a cumulative contribution rate of 90% are used for analysis.

Each principal component load can be written as:


(5) $$\begin{array}{r} l_{ij}=\sqrt{\lambda_{i}}e_{ij},\;\text{i},j=1,2,\cdots \text{p} \end{array}$$

The mathematical calculation of the comprehensive score of each sample reads:


(6) $$\begin{array}{r} F=\alpha_{1}z_{1},\alpha_{2}z_{2},\alpha_{3}z_{3}+\ldots \end{array}$$

In Equation (6), ***Z***_**s**_ is the principal component, **α**_**s**_ represents the variance contribution rate corresponding to the principal component. The result of the principal component analysis is the input of BPNN.

The initialization of the BPNN model includes the determination of the number of network layers, neurons in each layer, and the initial weight and threshold. The three-layer structure is selected for the teaching performance evaluation model. The number of neurons in the input layer equals the number of principal components by PCA. The structural block diagram of the evaluation model is shown in Supplementary Figure S2.

Sigmoid is used as the hidden layer function in the BPNN model. Thus, to avoid the saturation of the Sigmoid function, the input data must be normalized into (0,1) intervals. Here, the maximum–minimum method is used for normalization, and the mapminmax function in Matlab is called to realize this process. By default, mapminmax function normalizes the data into interval [−1,1].

## Questionnaire survey design and illustration of the surveyed target

### QS design

The subjects are the students of class 2 and class 9 of grade 55 Middle School in Xi'an. There are 39 students in class 2 and 42 students in class 9. Class 9 is the experimental class, and micro-video with interactive design is used as teaching resources. Class 2 is the control class, and micro-video without interactive design is used as a teaching resource. The pre-investigation of the two classes and the pre-test of autonomous learning ability reveal that the two classes have little difference in performance or autonomous learning ability. The same teacher teaches the two classes to avoid the influence of irrelevant factors on the experiment. The experiment is carried out in the No. 55 Middle School of Xi'an for eight consecutive weeks.

This experiment is based on quasi-experimental research methods, supplemented by QS. The specific experimental variables are as follows: (1) Independent variables are teaching resources. That is whether to use micro-video with interactive design as teaching resources to teach and learn. (2) Dependent variables include learning effect (activity performance, knowledge test, and autonomous learning ability) (3) Control variables include the number of students in two classes is equal. Importantly, there is no significant difference in the self-regulated learning ability of the original basic learning level. The same teacher teaches the two classes in the same teaching environment. The teaching progress is the same. The same teaching content is selected to carry out the same class hour teaching. The teaching evaluation criteria are consistent with the evaluation tools used.

In order to analyze the teaching results and verify the system's effectiveness, a test paper is designed to test students' mastery of knowledge points. There are eight questions in the test paper, including five multiple-choice questions, two calculation questions, and one simple answer. After the test, the corresponding curriculum effect evaluation is distributed to students, including student's name, course content, wrong knowledge points, classroom satisfaction, content acceptance, test satisfaction, and method validity. The last four items adopted a five-point system, that is, very unsatisfactory scores – 1, unsatisfactory scores – 2, average scores – 3, very satisfactory scores – 4, and very satisfactory scores – 5.

### Illustration on the experimental target

Two-hundred students from three classes in a university are selected as the research subjects. The experimental targets are divided into experimental group and control group. Under the same learning level, as usual, the control group adopts the traditional teaching method, and the experimental group adopts the teaching mode constructed here. After 3 months of teaching, students will finish the examination in 45 min in accordance with the requirements of the conventional examination during free time at night. Supplementary Figure S3 signifies the sample structure and respondents' basic information.

The survey lasted for 3 months from October to November of 2019, and the questionnaire is pre-surveyed, recycled, and improved. Large-scale analysis and research are conducted in December of the same year. A total of 200 questionnaires are distributed and 200 are recovered, with a recovery rate of 100%. However, 13 questionnaires are invalid and incomplete. One hundred eighty-seven questionnaires are valid, accounting for 93.5%. In order to verify the reliability, stability, and index system of the questionnaire data, the questionnaire quality is tested, and the α reliability coefficient is evaluated by SPSS 24.0 software. QS design, distribution, and data collection will not violate any personal privacy, which is carried out with the consent of the participants. QS has been approved by school leaders and relevant departments. These QS use an anonymous system, and the obtained data are only used for academic research. The questionnaire designed here does not involve students' personal privacy, which is agreed by the students in the school.

## Systematic test and analysis of teaching

### Comparison between system stress test results and system performance

According to the actual needs, the proposed system is tested twice, and the test results are shown in Supplementary Figure S4.

Supplementary Figure S4 indicates that when the number of online users is 10, the response time of the system for the first experiment is 1.6 s, while that for the second experiment is 1.8 s. When the number of online users is 100, the response time of the system in the first experiment is 3.6 s, while that in the second experiment is 4.1 s. When the number of online users reaches 200, the first response time is 5.5 s, while the second response time is 6 s. The overall data change trend shows that the response time of the proposed micro-video teaching platform increases with the increase in the number of online users. When the number of online users peaks, the response time of the system is 6 s, and no abnormality has occurred. Thus, the stress resistance of the proposed system is desirable.

The system comparison test results are shown in Supplementary Figure S5.

Supplementary Figure S5 implies that when the number of onliners is 10, the micro-video playback fluency of the previous micro-video teaching platform is 87%, while that of the proposed micro-video teaching platform is 90%. When the number of onliners reaches100 and 200, the playing fluency of the previous micro-video teaching platform is 90 and 89%, respectively. By comparison, the playback fluency of the proposed micro-video teaching platform is 82% under 200 onliners. With the increasing number of onliners from 10 to 200, the playback fluency of the system has somehow fluctuated, but the overall trend is downward.

### Algorithm performance test results

Further, the distance binary tree support vector machine (DBT-SVM) algorithm, SVM algorithm, and BT-SVM algorithm are employed respectively to classify the experimental data in the performance evaluation scale of the teaching platform. Supplementary Figure S6 displays the accuracy of the DBT-SVM algorithm, SVM algorithm, and BT-SVM algorithm, as shown in Supplementary Figure S6.

Supplementary Figure S6 demonstrates that the prediction accuracy of the DBT-SVM algorithm is higher than that of SVM and BT-SVM algorithms. The main reason is that the DBT-SVM algorithm adopts the strategy of generating a complete binary tree, so that the easily classifiable classes are separated first, avoiding the accumulation of errors, and thus improving the division accuracy.

Supplementary Figure S7 displays the algorithm test and training time.

Supplementary Figure S7 suggests that when tested under data1, data2, and data3, the SVM algorithm consumes a longer time compared with the DBT-SVM algorithm and BT-SVM algorithm, while the time consumption of the BT-SVM algorithm and DBT-SVM algorithm in the classification process is not much different. However, the difference between the BT-SVM algorithm and the DBT-SVM algorithm will continue to increase with the continuous increase in the amount of data. In short, the proposed DBT-SVM algorithm is feasible to evaluate the quality of classroom teaching in colleges and universities.

### QS results

The QS results are shown in Supplementary Figure S8. Supplementary Figure S8a shows the QS results of students' satisfaction with the micro-video course. Orange in the figure represents the students' satisfaction with the classroom before the proposed teaching system is used, and green denotes the students' satisfaction with the classroom after the proposed system is used. Supplementary Figure S8b displays the QS results of students' recognition of the micro-video classroom compactness. Orange in the figure represents students' recognition of classroom rhythm before the proposed teaching system is used, and green indicates students' recognition of classroom rhythm after the proposed system is applied.

Supplementary Figure S8a shows that before the proposed system is used, students' satisfaction with the classroom is 60%, and after the proposed micro-video teaching platform is applied, students' satisfaction with the classroom is 75%, with an increase of 15%. Similarly, before and after the proposed system is applied, the student's dissatisfaction with the classroom is 10% and 5%, respectively, which has been reduced by 5%. Hence, the proposed system improves students' satisfaction with the teaching classroom. Supplementary Figure S8b indicates that about 75% of students feel that classroom teaching becomes compact after the micro-video teaching platform is applied. It shows that the students who use the proposed micro-video teaching platform are very interested in the course, while the number of students who feel bored in the course is decreasing, thus proving that the proposed system has a good teaching effect.

In the 200 questionnaires returned, the accuracy of students' choice questions is shown in Supplementary Figure S9, and the accuracy of calculation questions and summary answers is shown in Supplementary Figure S10.

Supplementary Figure S9 signifies that the correct rate of the first choice of the experimental group is 92%, and that of the control group is 87%. The correct rate of the second multiple-choice test is 96% in the experimental group and 93% in the control group. The accuracy of the third-choice question is 91% and 88%. Overall, the accuracy of the experimental group students' multiple-choice answers is higher than that of the control group, which can explain that the teaching mode constructed here can help students learn better, can also better improve the accuracy of students' choice answers, and is conducive to improving students' scores.

Supplementary Figure S10 denotes that the accuracy rates of the first calculation questions in the experimental group and the control group are 96 and 87%, respectively. The correct rate of the second calculation is 88% in the experimental group and 90% in the control group. The accuracy of the third calculation question is 93 and 88%, respectively. The accuracy of students' answers in the experimental group is significantly higher than that in the control group. However, the combination of error-test teaching method and flow teaching method based on deep learning theory can improve the accuracy of students' calculation and short answer questions, which is higher than that of multiple-choice questions. The reason is that the calculation and short answer questions can show students' comprehensive ability, while the choice questions have certain guessing components.

Supplementary Figure S11 presents the scores of multiple-choice questions in the experimental group and the control group.

Signal ^*^ represents a significant difference, while ^*^^*^ represents a very significant difference. Supplementary Figure S11 reveals that there is a very significant difference in the accuracy of the students in the experimental group and the control group in answering multiple-choice questions 1, 2, 4, and 5 (*p* < 0.01), but there is no very significant difference in the accuracy of the students in the experimental group and the control group in answering multiple-choice questions 3 and 6 (*p* > 0.05).

### Comparison test of the system performance

The system test diagram before improvement is shown in Supplementary Figure S12a, and the system test diagram after improvement is shown in Supplementary Figure S12b.

Supplementary Figure S12a shows the test results of the traditional teaching system. The active interaction curve and the passive interaction curve of the traditional teaching system have no obvious fluctuation, indicating that the interaction of the traditional teaching system is not strong, the ability of active feedback information is poor, and there is no positive feedback from the user's questions. Supplementary Figure S12b presents the test results of the teaching system constructed here. The number of information submissions and information feedbacks in the teaching system is relatively close, and there is little change in the whole experimental process. The active interaction and the passive interaction curve have large fluctuation, which can explain that the teaching system constructed here has more frequent interaction, and the teaching information can get real-time and active feedback. The system can stimulate students' learning desires.

### Test results of the satisfaction with the system

Supplementary Figure S13 shows the test results of students' satisfaction with the teaching system constructed here.

Supplementary Figure S13 shows that under other teaching systems, students' satisfaction with the course is 60%. By comparison, under the proposed teaching system, students' satisfaction with the course is 75%, a 15% increase. Meanwhile, students' dissatisfaction with the course is 10% under other teaching systems, compared to 5% under the proposed teaching system. Thus, the proposed teaching system improves students' satisfaction with classroom teaching.

### Analysis of system baseline test results

The performance test aims to test the system requirements and performance objectives in the design process. The performance test considers the system response time, the calculation accuracy, and data security. At the same time, the performance bottleneck in the resource service platform of the school is understood to guide the optimization of platform expansion.

In the performance test of the school resource service platform, the users are selected to log in to the online micro-video teaching system, upload files, query resource files, add comments, and then exit the system. The test process uses the LOADRUNNER (an automated performance test software) to initialize 10 users. Then, it increases virtual users on the platform at the rate of five users/two seconds until virtual users reach100. Then, the performance test completes. Based on these, Tables 2–4 list the test results with 10 users, 30 users, 50 users, 80 users, and 100 users for system login, uploading, and querying.

**Table 2:** Test results of system login service performance.

**Client concurrency**	**Iteration times**	**Average response time**	**Success times**	**Disconnection response times**	**Data query failure times upon successful connections**	**Central Processing Unit (CPU) occupancy rate**	**Random Access Memory (RAM) occupancy (MB)**
10	10 × 20	0.46 s	10 × 20	0	0	1.1%	55
30	30 × 20	0.28 s	30 × 20	0	0	2.6%	56
50	50 × 20	0.23 s	50 × 20	0	0	2.2%	61
100	100 × 20	0.34 s	100 × 20	0	0	3.3%	65

Table 2 indicates that when the number of clients is 10–100, and each client simulates 20 virtual system logins to submit services, the system response time of each service is within 500 ms, the success rate is 100%, the CPU occupancy rate is 1.1–3.3%, and the RAM occupancy is 55–65MB.

Table 3 uses a 1G video file to test resource uploading performance, and each user tries to upload the resource file twice. The test results show that the average response time for 1G resources is <250 s, and the upload speed is about 4 m/s. Thus, the proposed teaching system's performance basically meets the online video teaching requirements.

**Table 3:** Resource uploading performance test.

**Client concurrency**	**Iteration times**	**Average response time**	**Success times**	**Disconnection response time**	**Data query failure times upon successful connections**	**CPU occupancy rate**	**RAM occupancy (MB)**
10	10 × 2	210 s	10 × 2	0	0	3.1%	231
30	30 × 2	214 s	30 × 2	0	0	3.6%	261
50	50 × 2	231 s	50 × 2	0	0	5.2%	298
100	100 × 2	248 s	100 × 2	0	0	4.3%	281

**Table 4:** Resource querying performance test.

**Client concurrency**	**Iteration times**	**Average response time**	**Success times**	**Disconnection response time**	**Data query failure times upon successful connections**	**CPU occupancy rate**	**RAM occupancy (MB)**
10	10 × 20	1.11 s	10 × 20	0	0	2%	151
30	30 × 20	1.14 s	30 × 20	0	0	3.6%	161
50	50 × 20	1.23 s	50 × 20	0	0	4.2%	162
100	100 × 20	1.48 s	100 × 20	0	0	4.3%	164

Apparently, the system response time for querying the file is within 1.5 s, the success rate reaches 100%, the CPU occupancy rate is below 5%, and the RAM occupancy is relatively high. With 2,000 concurrent queries, the RAM occupancy reaches over 160 MB.

## Conclusions

Here, a comprehensive investigation is conducted on the teaching modes of a specific subject, the disadvantages of the current teaching modes of colleges and universities are pointed out, including the single teaching mode and insufficient teaching resources. Thereupon, a deeper understanding is formed on the research and application status of remote micro-video teaching. Afterward, the micro-video teaching platform is constructed, the system requirements, nonfunctional requirements, and system feasibility of the teaching platform are analyzed in detail, and the use-case analysis is carried out for three different types of platform users: teachers, students, and system administrators. Further, the overall framework of the micro-video teaching platform, user information management, and database of the micro-video teaching platform system is designed and explained. Meanwhile, a teaching quality evaluation system based on the DBT-SVM classification method is established using the SVM algorithm and DBT algorithm, through which the teaching quality of the proposed micro-video teaching platform is evaluated. The results show that the proposed micro-video teaching platform has good performance and stability. Still, there are some shortcomings. At present, the proposed system is only applicable to the teaching of a small number of courses, and the design of the micro-video teaching platform is not mature enough and needs improvement. In the follow-up, the application scope of the proposed system will be further expanded.

## Data availability statement

The raw data supporting the conclusions of this article will be made available by the authors, without undue reservation.

## Ethics statement

The studies involving human participants were reviewed and approved by Zhejiang Normal University Ethics Committee. The patients/participants provided their written informed consent to participate in this study. Written informed consent was obtained from the individual(s) for the publication of any potentially identifiable images or data included in this article.

## Author contributions

All authors listed have made a substantial, direct, and intellectual contribution to the work and approved it for publication.

## Funding

This study was supported by a grant 18CJY26 from the project of the Shandong Social Science Plan in 2018, the 2019 Higher Education Teaching Reform Project of Guangdong Province: Research on Teaching Design and Teaching Methods of Urban Design Course Integrated with Ideological and Political Work, and the School-Leveled Teaching Case Database for Professional Degree Postgraduates in 2022.

## Conflict of interest

The authors declare that the research was conducted in the absence of any commercial or financial relationships that could be construed as a potential conflict of interest.

## Publisher's note

All claims expressed in this article are solely those of the authors and do not necessarily represent those of their affiliated organizations, or those of the publisher, the editors and the reviewers. Any product that may be evaluated in this article, or claim that may be made by its manufacturer, is not guaranteed or endorsed by the publisher.
