# Supplementary material for: Exploration of micro-video teaching mode of college students using deep learning and human–computer interaction
Source: Front Psychol. 2022 Sep 2;13:916021. doi: 10.3389/fpsyg.2022.916021 (PMC9478761; doi:10.3389/fpsyg.2022.916021)
Supplement: Supplementary file 1 [file Data_Sheet_1.docx]

**Supplementary Material**

Supplementary Figure 1 BPNN structure

Supplementary Figure 2 Model structure diagram

Supplementary Figure 3 Basic situation of the interviewees (A. proportion of gender; B. proportion of classes)

Supplementary Figure 4 System stress test results

Supplementary Figure 5 Micro-video playback fluency test results

(a) data1 (b) data2

(c) data3

Supplementary Figure 6 Comparison of algorithm accuracy

(a) data1 (b) data2

(c) data3

Supplementary Figure 7 Comparison of algorithm consumption time

(a) QS results of students' satisfaction with micro-video classroom (b) QS results of students' recognition of the micro-video classroom compactness

Supplementary Figure 8 QS results

Supplementary Figure 9 Statistical analysis of the multiple-choice questions

Supplementary Figure 10 Statistical analysis of the accuracy of the calculation questions and summary questions

(a) (b)

Supplementary Figure 11 Score comparison (a: score values of experimental group and control group; b: t and p values of the experimental group and the control group)

(a) algorithm test before improvement (b) algorithm test after improvement

Supplementary Figure 12 Results of systems comparison test

(Note: because the variables in the improved algorithm are greatly disturbed by the external environment, the curve of the experimental results deviates to a certain extent, but the error is within the allowable range)

Supplementary Figure 13 Students' satisfaction with the teaching system
